# Effectiveness of resistance training on resilience in Hong Kong Chinese older adults: study protocol for a randomized controlled trial

**DOI:** 10.1186/s12877-021-02197-8

**Published:** 2021-04-15

**Authors:** Pak-Kwong Chung, Chun-Qing Zhang, Yanan Zhao, Ming Yu Claudia Wong, Chun Hu

**Affiliations:** 1 Department of Sport, Physical Education and Health, Hong Kong Baptist University, 224 Waterloo Road, Kowloon Tong, Kowloon, Hong Kong, China; 2 Department of Psychology, Sun Yat-sen University, Guangzhou, China; 3 School of Sports Science and Physical Education, Nanjing Normal University, Nanjing, China; 4 Student Mental Health Education Center, Northwestern Polytechnical University, Xi’an, China

**Keywords:** Resilience, Older adults, Resistance training, Ageing, Psychological health

## Abstract

**Background:**

There is on one hand sufficient evidence showing strong association between resilience and self-rated successful aging. On the other hand, strength training could contribute the cultivation of resilience among older adults. Therefore, the current study aims to examine the effectiveness of resistance training on resilience among Chinese older adults in Hong Kong.

**Methods:**

This study will apply a three-group, double blinded (outcome assessors and data analysts), randomized controlled trial (RCT) to examine the effectiveness of the interventions on resilience, functional fitness, and health related quality of life immediately after a 16-week intervention, as well as the residual effects 12 weeks after completion of the interventions.

**Discussion:**

It is expected that resistance training is promising or even superior to aerobic training in the improvement of resilience. Given the limited evidence on the literature, it is urgently needed to explore the effects of resistance training on the improvement of resilience in older adults. Findings of the current study can contribute to the development of effective resistant training programs for the promotion of resilience among older adults.

**Trial registration:**

The trial is registered at the ClinicalTrials.gov PRS (Trial ID: NCT04690465; Date of First Posted: 30/12/2020).

## Background

With support from the health and medical research as well as technological advancement, the older population in the world has grown dramatically over the last several decades [1]. A similar situation was reported in Hong Kong. As of 2015, there were approximately 7.346 million people living in the city with a total of 1105 km^2^ of land. Among the 7.346 m population, 16% (1.17 m) was older adults (65 and over) [2], which has given Hong Kong many challenges in dealing with problems caused by ageing. Among the challenges, maintaining health related quality of life in the later years and reducing health related medical cost would be of great importance for both older individuals and the society. According to Rose and colleagues [3], aging is defined as a decline or loss (a “de-tuning”) of adaptation with increasing age, caused by a time-progressive decline of Hamilton’s forces of natural selection. Traditionally, older age has been viewed negatively as a time of frailty, disability, declining function, and greater physical and mental limitations. However, many older adults actually experience high wellbeing and quality of life and consider themselves to be aging successfully despite the onset of chronic conditions. It is believed that the combined psychological and physical characteristics of high resilience have the potential to facilitate the process of successful aging [4].

There is sufficient evidence showing strong association between resilience and self-rated successful aging [5, 6]. Resilience plays a critical role when older adults face the daily stress that comes from physical and mental loss with advancing age (e.g., changes in cognitive functioning, aging-related degradations in physical fitness), dysfunctional relationships with family members, increased isolation from society, and even the impact of extreme weather conditions [7]. There is considerable divergence in the literature in terms of construct and operational definitions of resilience. The focal point is whether resilience is a trait, an outcome or a process. During the past decades, some researchers held the view that resilience incarnates the personal qualities that empower individuals to thrive in the face of challenging circumstances. Resilience can be defined as the ability to bounce back or recover from stressful experiences, wherein ‘resilience’ derives from the Latin words salire (to leap or jump), and resilire (to spring back) [8]. Some scholars held the view that resilience as an outcome of successful adaptation despite challenging or threatening circumstances [9], or a class of phenomena characterized by good outcomes in spite of serious threats to adaptation or development [10].

In recent years, as research involved in the field increases, a growing number of researchers believe that resilience is a dynamic process encompassing positive adaptation within the context of significant adversity [11], which indicates that resilience is based on the balances between risk factors and protective factors. The American Psychological Association [12] defines resilience as the process of adapting well in the face of adversity, trauma, tragedy, threats or significant sources of stress — such as family and relationship problems, serious health problems or workplace and financial stressors. Current evidence supports viewing resilience as a universal dynamic process that helps individuals navigate their way in the context of exposure to stress using physical, psychological, social, and cultural resources that sustain their well-being [13] or an adaptive process that benefits maintaining, or swiftly regaining, homeostasis in conditions of stress [14].

Considering the significant meaning of resilience to older adults’ daily life, promotion of resilience among older adults is certainly worthy of lucubration. The promotion is even more urgent to Hong Kong given that one in three people will be 65 years old or above by 2036 [15]. Among the various influential factors on resilience, health-related lifestyles and higher functionality of individuals are identified as having strong associations with high resilience [16]. Moreover, regular exercise and/or physical activity (PA) has been found to be the key mediator within these associations [17]. Except for the well-established physiological benefits, psychological positive changes brought from resistance training should not be ignored. Recent evidence revealed the connections between resistance training and self-efficacy as well as social interaction among older adults [18]. During a 12-week program of moderate-to-high intensity resistance training, older adults reported a “good feeling” from doing resistance training. The “good feeling” might come from two facets. Firstly, resistance training is often stereotyped as being a young persons’ activity, many older adults are not educated in correct way to do resistance training. However, the older adults found out that they are not too old and stupid to do resistance exercise. They recognized their capabilities and showed improvement in self-efficacy. Secondly, the older adults might view the resistance training as part of the real life and gradually get a feeling of “taking control” of their life after repeatedly increased in workload during training. Furthermore, resistance training produced more training efficacy on increased resistance from the perspective of physiological stimulations.

Strength training was found to increase muscle strength by increasing muscle mass, and by improving the recruitment of motor units as well as increasing their firing rate in older adults. It was recommended that healthy old people should train 3 or 4 times weekly for the best results [19]. In a preliminary review, Windle [20] found a lack of publications that had adopted an intervention design in the studies of resilience in older adults. He further recommended that studies employing randomized controlled trial (RCT) design with specific intervention strategies as well as less robust evaluation using controlled trials should be conducted. MacLeod and colleagues [4] also found that the resilience interventions targeting older adults generally were not existing. They also found no tested interventions incorporating physical activity to build resilience among older adults, despite research suggesting that PA is an important factor of resilience.

Considering the above evidence, we may expect that resistance training is promising or even superior to aerobic training in the improvement of resilience. However, little evidence is found based on the literature and therefore it is a need to explore the effects of resistance training on the improvement of resilience in older adults. Based on the evidence of Windle [20] and MacLeod and colleagues [4], as well as the potential benefits of strength training contributes to the older people as indicated by Mayer and colleagues [19], the current study, adopting a RCT design, aims to examine the effectiveness of resistance training on resilience among Chinese older adults in Hong Kong.

## Methods

### Study design

This study will apply a three-group, double blinded (outcome assessors and data analysts), randomised controlled trial (RCT) to examine the effectiveness of the interventions on resilience, functional fitness, and health related quality of life immediately after a 16-week intervention, as well as the residual effects 12 weeks after completion of the interventions. The double blinding adopted in this study is to reduce the bias and to ensure that those who are collecting the outcome measures (such as research assistants collecting data via questionnaires and fitness assessors collecting data from the functional fitness test), and those who are inputting and analysing the data, have no knowledge regarding groups allocation status of the study participants [21].

In assigning the groups, the CONSORT procedure will be followed [22]. The three groups will be resistance training group, Tai Chi group, and control group. Tai Chi will be selected as an active concurrent control group for adding value in the comparison. The reason for choosing Tai Chi is because it has been proven to help the heart-failure patients improve their self-efficacy and self-esteem of resilience measured by the Resilience Scale [23]. Also, the aerobic type of Tai Chi will also demonstrate a bigger contrast to the anaerobic type of resistance training and therefore, the comparison on the training effects between resistance training and Tai Chi will add value to selection of exercise for future training and intervention of further study. Ethical approval from the Research Ethics Committee of the Hong Kong Baptist University has been obtained.

### Participants

The participants will be recruited from community senior service centers in Hong Kong by the approach of convenience sampling. Currently we are working with more than 30 community senior service centers locating in 18 districts in our ongoing research projects, in which a strong and supportive collaboration has been established for our recruitment of participants. The eligibility criteria for selection of subjects include: (1) 65 to 74 years old; (2) capable of walking without assistive device; (3) apparently healthy and live independently in communities; (4) no cardiovascular or related diseases that prevent from resistance training; (5) pass the PAR-Q screening or with physician’s advice on readiness of participation in resistance training; (6) no substantial experience in practicing resistance training or Tai Chi; and (7) low-to-moderate level of resilience. Participants with cognitive impairment, which will be determined by using the Chinese version of the Mini-Mental Status Examination (i.e., score < 24) [24] will be excluded. All participants will be recruited following the principle of voluntariness and their data will be kept confidential. Informed consent letters will be signed by the qualified participants prior to the commencement of intervention.

### Sample size estimation

Based on the previous intervention studies, the most frequently reported effect size was ranged from moderate (0.30) to high (0.70) [25–29], a moderate effect size at 0.30 will be adopted for the current study. To ensure an 80% probability for detecting a treatment difference at a two-sided 5% level of significance and taking into account a 20% of potential dropout rate [30], a sample size of 40 participants per group (totally 120 for 3 groups) will be required in the current study.

### Grouping and randomization

Qualified participants who have signed a consent letter will be randomly assigned into three groups by a draw of lots, in a ratio of 1:1:1. The three groups will be 1) Intervention group: Resistance training (*n* = 40); 2) Active concurrent control group: Eight-form Yang-style Tai Chi program (n = 40); and 3) Non-treatment control group (n = 40). The CONSORT flow diagram is as follow:

### Training program design and implementation

With the support of the Physical Fitness Association of Hong Kong, China (PFA) and the Hong Kong Wushu Union (HKWU), it would expect that both resistance training and Tai Chi programs will be delivered safely and effectively by qualified coaches with professional monitoring.

#### Exercise prescription

Generally, an effective exercise program is conducted 3 times per week for 3 months (12 weeks) [30]. In considering the participants who are a group of older adults without regular exercise experience may need more time to adapt to regular exercise, a longer duration by extending the intervention period to 4 months (16 weeks) will be adopted in this study. With 3 sessions per week, the duration of each session will be 60 min, which include 10 min of warm-up, 40 min of main exercise, and 10 min of cool-down. The intensity will be light to somewhat hard (RPE 11 to 13; using the Cantonese version of RPE) [31]. In resistance training, the weights (resistance) will be from participants’ own bodies, dumbbells, and adjustable ankle weights.

The simple equipment and forms of training selected in the current study is for sustainability of training after the intervention. The participants could continue their training at homes or at the senior centers as no sophisticated equipment required for the training. The dumbbell weights used in the resistance training program are 1 kg, 2 kg, 3 kg, and 4 kg for women and 2 kg, 3 kg, 4 kg, 5 kg for men, and the adjustable ankle weights are from 1 kg to 10 kg, based on the reference of the book “*Growing Stronger: Strength Training for Older Adults”* [32] as well as advice of PFA. The intensity of each exercise will be 3–4 sets with 10–12 repetitions per set. Adjustment will be made in accordance with individual’s level of fitness as well as adaptability and progression of the training. The improvement will be on progressive and safe manner under proper guidance of the coach. Each participant will be provided a log-book for recording the intensity, progression, and personal feedback of training for evaluation during and after the training.

In Tai Chi, the 16-week program will be divided into cognitive, associated, and automatic stages. The coach will apply a group teaching with individual instruction on specific movements based on participant’s needs in skills learning and acquisition. The same training principles of individuality and progression as well as training log-book used in resistance training will also be applied to Tai Chi training program. In order to achieve a safe and effective teaching and learning, both resistance training and Tai Chi groups will be randomly divided into two sub-groups, with 20 participants in a sub-group. It means that 2 sub-groups of resistance training as well as 2 sub-groups of Tai Chi training, which will be conducted in separate timeslots but taught by the same coach of the program (one coach for resistance training, and one coach for Tai Chi training) at the same venue so as to ensure both sub-groups will receive similar teaching and learning quality.

#### Intervention group (resistance training)

Participants will receive a 16-week resistance training program, with 3 times a week (a total of 48 training sessions) and 60 min per session intervention, which is adopted from the 12-week resistance training program introduced in the book “*Growing Stronger: Strength Training for Older Adults”* (Seguin et al., 2002), with minor modifications under the advice of PFA. Below is the sketch of the resistance training program which will be taught by the qualified resistance training coach recommended by PFA (see Table 1).

**Table 1:** Training program for the strength training group

Strength Training	Warm-up (10′)	Exercises (40′): 3–4 sets with 10–12 repetitions per set	Cool-down (10′)
**Part I (week 1–2)** To strengthen body slowly and gently using only own body weight	light walking and stretching	Six exercises: squats, wall push-ups, toe stands, finger marching, pelvic tilt, floor back extension	light walking and stretching
**Part II (week 3–6)** To strengthen body slowly and gently, using own body weight and light dumbbells	light walking and stretching	Eight exercises: squats, wall push-ups, toe stands, finger marching, pelvic tilt, floor back extension, biceps curl, overhead press	light walking and stretching
**Part III (week 7–10)** To increase exercise to 10, and add adjustable ankle weights	light walking and stretching	Ten exercises: squats, wall push-ups, toe stands, finger marching, pelvic tilt, floor back extension, biceps curl, overhead press, knee extension, knee curl	light walking and stretching
**Part IV (week 11–16)** To increase exercise to 12, with less resting time between sets	light walking and stretching	Twelve Exercises: squats, wall push-ups, toe stands, finger marching, pelvic tilt, floor back extension, biceps curl, overhead press, knee extension, knee curl, step-ups, side hip raise	light walking and stretching

#### Active concurrent control group (tai chi)

The active concurrent control group will receive Eight-form Yang-style Tai Chi program which has been confirmed feasible and will be taught by the qualified coach recommended by HKWU (see Table 2).

**Table 2:** Training program for the Tai Chi group

Tai Chi	Warm-up (10′)	Exercises (40′)	Cool-down (10′)
**Part I (week 1–4)** Cognitive stage	light walking and stretching	**Learn the following movements with the coach guidance**: Repulse Monkey, Brush Knee Twist Step, Part Wild Horse’s Mane, Wave Hands in Clouds, Golden Rooster Stand on one Leg, Heel Kick, Grasp Swallow’s Tail, and Cross Hand	light walking and stretching
**Part II (week 5–10)** Associated stage	light walking and stretching	**Refine all the movements with supervision.**	light walking and stretching
**Part III (week 11–16)** Automatic stage	light walking and stretching	**Play all the movement by oneself smoothly.**	light walking and stretching

#### Non-treatment concurrent control

Participants in this group will not participate in any specific intervention during the whole study (the 16-week intervention and 12-week follow-up periods), but they will be asked to keep a daily log on their physical activity, medicine used, illness, diet, sleep quality and other health and physical activity related information (e.g., attending healthy eating workshops). In addition, participants of the control group will be asked to report to the research assistant (RA) if a major change has been made in the aforementioned aspects. The RA will also check the daily logs of the participants through telephone every 2 weeks. Data from those who had changed their normal lifestyles (especially taking up regular physical activity) will be examined for exclusion in the subsequent data analysis.

### Outcome measures

#### Primary outcome – resilience

In order to meet the needs of the local older population, the Chinese version of RS (CRS) [33] will be used to measure participants’ resilience in the study. The CRS was modified from the Resilience Scale developed by Wagnild and Young [34] with subsequent validation in Chinese older population and confirmed its four-factor structure: equanimity, meaningfulness, ceaseless self-improvement, and self-reliance. A 7-point Likert scale from 1 (highly disagree) to 7 (highly agree) is used, with a greater score mirroring higher level of resilience. Three levels are set, with scores of 145 and above indicating moderate to high resilience, scores from 126 to 144 indicating low to moderate levels of resilience, and scores less than 126 indicating low resilience. The internal consistency (Cronbach’s α) for the total scale was 0.95, and the test–retest reliability was 0.80 [33].

#### Secondary outcomes: functional fitness

The functional fitness (FF) of participants will be measured using the Senior Fitness Test (SFT) battery [35]. The SFT is a widely used measurement for functional fitness of older adults [36] in the ageing and physical activity area. It was first developed and validated by Rikli and Jones [37] with the purpose of early identification of older individuals at risk for losing functionality. There are 7 testing items assessing all the 5 dimensions of functional fitness, including body mass index (BMI), 30s chair stand for lower limbs’ muscle strength, 30s arm curl for upper limbs’ muscle strength, 2 min step test for aerobic endurance, chair sit-and-reach test for lower body flexibility, back scratch test for upper body flexibility, and 8 ft. up-and-go test for mobility and dynamic balance [37]. Rationales behind the SFT along with the validity and reliability of these testing items have been well described in Senior Fitness Test Manual [35]. The present study will strictly follow the testing procedures as suggested by this manual. The testing team supported by PFA will be invited to conduct the test. The testing team was trained to complete the SFT for about 1000 older adults in our previous study for establishing FF norms for Hong Kong older adults [38]. All the tests will be conducted before (pre-test) and after (post-test) the 16-week interventions. In order to examine how long the training effect might persist, a follow-up test will be carried out at beginning of the13^th^ week, 12 weeks after completion of the intervention (follow-up test).

#### Secondary outcomes: health related quality of life

The Short Form-36 (SF-36) is a widely used health survey questionnaire, especially for older adults to assess the health related quality of life (HRQoL) [39]. The SF-36 consists of 36 items covering two dimensions—physical health (physical functioning, role limitations due to physical problems, role limitations due to emotional problems, and social functioning) and mental health (mental health, vitality, body pain, and general health perception). In the current study, the Chinese version of the SF-36 (C-SF-36) will be used. The C-SF-36 was translated and validated by Li and colleagues [40], which has demonstrated satisfactory test–retest reliability (r = 0.66–0.94). In our previous study using the C-SF-36 as the measure for the older adults [41], we found that the Cronbach alpha was 0.846 for the physical dimension and 0.837 for the mental dimension of quality of life, indicating that the Chinese version of the SF-36 has high convergent validity.

### Data analysis

Descriptive statistics will be used to describe baseline characteristics such as age, body weight, gender, resting heart rate, living status (alone or with family) of the participants in the study. The primary outcomes (resilience scores) will be analysed using an intention-to-treat (ITT) approach, and a sensitivity test will subsequently be conducted using the available data. Missing data will be replaced using the last observation carried forward method. For the secondary outcomes, per-protocol analysis will be conducted using the available data. The statistical significance level was set at *p* < .05. All data will be analyzed using SPSS Version 24.0 (IBM, Chicago, IL).

For the within group test, a one-way repeated measure analysis of variance (ANOVA) will be applied to each group to determine the changes of each outcome parameter among the three time points (i.e., pre-test, post-test, and follow-up test). When the sphericity assumption was violated, the Greenhouse–Geisser correction will be used when epsilon < 0.75, or the Huynh–Feldt correction will be used when epsilon > 0.75 [42]. Post hoc analysis with Bonferroni correction will be conducted to explore the differences between the pre-test and post-test, as well as post-test and follow-up test. The mean differences with 95% confidence intervals (CIs) will be reported. For the inter-group test, a one-way analysis of covariance (ANCOVA) with the baseline value as a covariate will be conducted at the post-test, and follow-up test to determine the group effect on each outcome parameter. Subsequently, the differences between the resistance training group and control groups as well as between the resistance training and Tai Chi groups will be analysed through planned contrasts. The effect size r was calculated manually according to r = sqrt [t2/(t2 + df)]. According to Cohen [43], r = .10, .30, and .50 represent a small, medium, and large effect size, respectively. The whole project is expected to complete within 18 months.

## Discussion

In their review study on the impact of resilience among older adults, MacLeod and colleagues [4] found from the studies that those older adults who were highly resilient are more physically active and likely to age successfully. This concept provides further support for the idea that physically active older adults may be able to maintain their health and adapt to the challenges of aging with greater success, and finally reduced mortality risk and increased longevity. Shephard [44] also indicated that among various ways to improve resilience, exercise should always be taken as the preferential way given its positive psychological and physiological benefits and minimal side effects. This is especially applicable to the older population. From a scoping review of the related literature, Waechter and Wekerle [45] found interventions with a mindful component (e.g., yoga, Tai Chi, and Qi Gong) may show particular promise in bolstering resilience. Similar findings were found among older population [23, 46, 47], indicating that resilience at least can be improved from the perspective of regular exercise.

Moreover, the underlying physiological mechanisms can help provide evidence for the effect of exercise on improving resilience. People who engage in more exercise and report better physical fitness appear to buffer against stress-related diseases owing to its blunting/optimizing effects on hormonal stress responsive systems, such as the hypothalamic–pituitary–adrenal axis and the sympathetic nervous system. This blunting appears to contribute to reduced emotional, physiological and metabolic re-activities as well as increased positive mood and well-being [17]. Another mechanism whereby regular exercise may confer resilience is through minimizing excessive inflammation. Chronic psychological stress, physical inactivity and abdominal adiposity have been associated with persistent, systemic, low-grade inflammation and exert adverse effects on mental and physical health [17]. The anti-inflammatory effects of regular exercise can promote behavioural and metabolic resilience and protect against various chronic diseases associated with systemic inflammation. Moreover, exercise may benefit the brain by enhancing growth factor expression and neural plasticity, thereby contributing to improved mood and cognition [48].

Considering the benefits of exercise or physical activity contributing to resilience of older adults, MacLeod and colleagues [4] found that the resilience interventions specifically targeting older adults generally were not existing. Most of the studies were targeting high-risk younger adults or military veterans at risk for suicide. They also found that most were academic research studies with methodological weaknesses, small numbers, no evaluations or outcome measures, and are not scalable or validated for older adults. The review also found no tested interventions incorporating physical activity to build resilience among older adults, despite research suggesting that physical activity is an important factor of resilience. Based on this critical comment, we found that there is an urgent need to fill the research gap by conducting intervention, with randomized control trial design to examine the effectiveness of physical training on resilience in older adults. In view of the forms of exercise or physical training in the previous studies that tended to be in aerobic type, such as Yoga, Tai Chi, and Qi Gong [23, 45–47], it may add value to this research topic if an anaerobic type of exercise or physical training program such as resistance/strength training could be used for the intervention. Overall, it is expected that findings of the current study could contribute to the development of effective physical training programs on improvement of resilience as well as fill the research gap in this important topic on promotion of acting ageing.

## Data Availability

Not applicable.

## Abbreviations

RCT: Randomised Controlled Trial
RPE: Rating of Perceived Exertion
RA: Research Assistant
CSR: Chinese Version of Resilience Scale
BMI: Body Mass Index
SF-36: Short Form-36
C-SF-36: Chinese Version of Short Form-36
ITT: Intention-To-Treat Approach
ANOVA: One-Way Repeated Measure Analysis of Variance
Cis: Confidence Intervals
ANCOVA: One-Way Analysis of Covariance
